# The Memory Superiority Effect of Induced Insight in Semantic Versus Perceptual Levels

**DOI:** 10.3390/jintelligence14050084

**Published:** 2026-05-13

**Authors:** Zhonglu Zhang, Yuxin Zeng, Wenkang Yu, Zeying Zheng

**Affiliations:** Department of Psychology, School of Education, Guangzhou University, Guangzhou 510006, China; zin0312@163.com (Y.Z.); 2112508038@e.gzhu.edu.cn (W.Y.)

**Keywords:** insight, visuo-perceptual level, semantic level, memory, retrospective confidence judgment

## Abstract

Previous studies have established that insight enhances memory across a variety of tasks. However, it remains unclear whether memory performance differs between perceptual and semantic insights. To address this issue, this study employed a learning–testing paradigm with Chinese riddles. In the learning phase, individuals judged whether they had grasped the relationship between each riddle and its solution (a Chinese character) under four conditions created by crossing match status (matched vs. unmatched) with riddle type (visual-rule vs. semantic-rule). During the testing phase, they performed immediate and delayed recognition tasks, judging whether each presented character was old or new. The results showed that, relative to trials on which riddles were not solved (primarily unmatched riddles), participants exhibited higher hit rates, recognition rates, and retrospective confidence judgment (RCJ) in both immediate and delayed recognition tasks when they experienced sudden insight into matched riddles. This reflects a memory advantage for insight induced by solution-appropriate processing. Moreover, hit rates, recognition rates, and memory confidence judgment accuracy were consistently higher for semantic insight than for perceptual insight, regardless of match condition (significant main effects with no interaction). In contrast, memory confidence judgment was higher for semantic (vs. perceptual) insight under matched conditions but not under unmatched conditions (significant interaction). Collectively, these findings suggest that insight yields better memory performance when it operates at the semantic level than at the perceptual level.

## 1. Introduction

Insight is often described as a sudden realization of the underlying solution or rule, involving both a cognitive component of restructuring and an affective component of “aha!” experiences (Stuyck et al., 2021; Wiley & Danek, 2024; Vitello & Salvi, 2023). Specifically, the cognitive component entails representational transformation through the integration of appropriate novel associations (Becker et al., 2025; Graf et al., 2023; Kounios & Beeman, 2014; Luo & Knoblich, 2007; Macchi et al., 2025; Zhang et al., 2024; Zhang et al., 2025a), whereas the affective component is characterized by the emotional “aha!” experiences that often accompany insight solutions (Bowden & Jung-Beeman, 2007; Kounios & Beeman, 2014; Su & Ye, 2025). Insight can occur at different levels. For example, solving camouflaged object riddles requires perceptual insight to identify the target object (e.g., Ludmer et al., 2011), whereas establishing remote associations at the semantic level is necessary for solving remote associate problems (Bowden & Jung-Beeman, 2007).

Previous research has consistently indicated that insight, as a distinct form of learning, improves memory relative to non-insightful learning across a range of tasks, including visual problems that rely on visual rules and verbal insight problems involving semantic rules. On the one hand, several studies have demonstrated a memory superiority effect of insight in visual problems (e.g., Kizilirmak et al., 2016b; Ludmer et al., 2011; W. Shen et al., 2021, 2022; Wills et al., 2000; Zhang et al., 2025c). In a study by Wills et al. (2000), participants sketched either incomplete dot patterns (insight condition) or complete patterns (non-insight condition) and then viewed the complete images after 5 s delay. The insight group showed better memory retention for the complete images than the non-insight group. Similarly, Kizilirmak et al. (2016b) examined participants’ recognition of blurred images before they were given the correct solutions, and recorded whether they experienced an “aha!” moment during the task. Follow-up memory tests revealed that spontaneous insight was associated with better memory than instructed solving, and that self-reported “aha!” feeling correlated with better recall. Using a similar blurred image task, Ludmer et al. (2011) found that participants exhibited higher confidence ratings for correctly recognized images than for incorrectly recognized images one week later. Shen et al. investigated the impact of creative advertisements on memory, comparing recall and recognition performance after 5 min and 3 days. The findings showed that individuals who reported insight exhibited better recall, recognition, unconscious memory, and higher retrospective confidence judgment than those who did not experience insight (W. Shen et al., 2021, 2022). Zhang et al. (2025c) used a visuo-perceptual Chinese character transformation task in which participants removed a visual component from a pseudo-character on the right and transferred it to a pseudo-character on the left to form two valid characters (solutions). Recognition memory for these solutions was tested after either 4 min or 24 h later. The to-be-removed component was either tightly or loosely integrated with the remaining part of the character, and participants showed better memory performance in the tight (insightful) condition than in the loose (non-insightful) condition.

On the other hand, some studies have confirmed the memory superiority effect of insight in verbal problems (e.g., Auble et al., 1979; J. Chen et al., 2023; Cui et al., 2021; Du et al., 2017; Kizilirmak et al., 2016a, 2019, 2021). For instance, Auble et al. (1979) demonstrated that memory retention was enhanced when riddle answers were presented after a delay (insight condition) compared to when answers appeared simultaneously with hints or incorrect hints (the non-insight condition). In studies by Kizilirmak et al. (2016a, 2019, 2021), participants completed solvable (sudden comprehension, insight condition) and unsolvable (continuous incomprehension, non-insight condition) remote associate tasks (RAT) followed by a recognition task. The findings indicated that sudden insight led to superior memory performance relative to non-insight conditions, as measured by both direct recognition and indirect problem-solving, in both young and old adults. Consistently, studies employing the Chinese version of CAT have shown that spontaneous insight (indicated by an “aha!” moment) yields more effective memory outcomes than non-insight (absence of an “aha!” moment), including faster response times (Cui et al., 2021; Du et al., 2017) and higher rates of correct repeated solutions (J. Chen et al., 2023). In summary, although prior research has established the memory superiority effect of insight at both the perceptual level (in visual problems) and the semantic level (in verbal problems), it remains unclear whether memory performance differs between semantic and perceptual insight.

Moreover, previous studies have indicated that the memory advantage of insight can be related to representational transformation through the formation of appropriate and novel associations (Becker et al., 2025; S. Chen et al., 2021; Zhang et al., 2025b; Zhao et al., 2025). Specifically, Zhao and colleagues investigated the memory effect of insight using the novelty-based definition of insight (by comparing novel vs. routine conditions). They discovered that appropriate and novel solutions were recalled and recognized more effectively than appropriate conventional ones (S. Chen et al., 2021; Zhao et al., 2025). This insight memory advantage was associated with prolonged gaze durations on novel solutions (Zhao et al., 2025) and enhanced activation in the right hippocampus, a region implicated in novelty processing (S. Chen et al., 2021). Consistently, Zhang et al. (2025b) found that participants had better memory for problem–solution associations in the high-novelty and appropriate condition than in the low-novelty and appropriate condition. Similarly, Ding et al. (2023, 2024) observed a positive relationship between subjective novelty and individuals’ performance recall and recognition performance for solutions from the Alternative Uses Task. Moreover, they found that this memory superiority effect was associated with activation of the right hippocampus and left medial prefrontal cortex (mPFC), indicating the role of forming appropriate and novel associations. Meanwhile, Kizilirmak et al. (2019) found that higher recognition rates of sudden comprehension (vs. those not incomprehension) in RAT were associated with activations of the hippocampus and left mPFC, two brain regions related to the integration of appropriate and novel associations (Milivojevic et al., 2015; Zhao et al., 2014). Consistently, Zhang et al. (2025d) found better memory performance for the comprehension of appropriate (vs. inappropriate) associations. Additionally, X. Wu et al. (2018) found the highest memory performance on solutions when participants solved chunk decomposition under conditions that were both highly novel and appropriate. Furthermore, Becker et al. (2025) provided comprehensive evidence that better long-term memory was predicted by enhanced activations and the functional connectivity of brain networks including ventral occipito-temporal cortex and hippocampus, indicating the role of representational restructuring via the integration of novel and appropriate information.

Taken together, previous studies have revealed a robust memory superiority effect of insight and clarified its underlying mechanisms across various insight problems (including visual and verbal problems). However, it remains unclear whether the memory benefit of insight differs between the semantic and perceptual levels. Specifically, prior work has shown that insights can emerge at different levels. Several studies have documented neural activation in the visual and parietal cortices, indicative of visuo-perceptual level representational reorganization during insight. These studies employed the visual Mooney identification task (Dolan et al., 1997; Ludmer et al., 2011) and the visual character decomposition task (Luo et al., 2006; Tang et al., 2016). Other studies have found elevated activation in the anterior superior and middle temporal gyrus (Jung-Beeman et al., 2004; Tik et al., 2018). Considering the fundamental role of the anterior superior/middle temporal gyrus in semantic processing (Binder & Desai, 2011), these findings suggested that semantic-level processing underlies certain forms of insight. This raised a critical unanswered question: Does the memory effect of insight differ between the semantic and perceptual levels?

**Figure 1:**
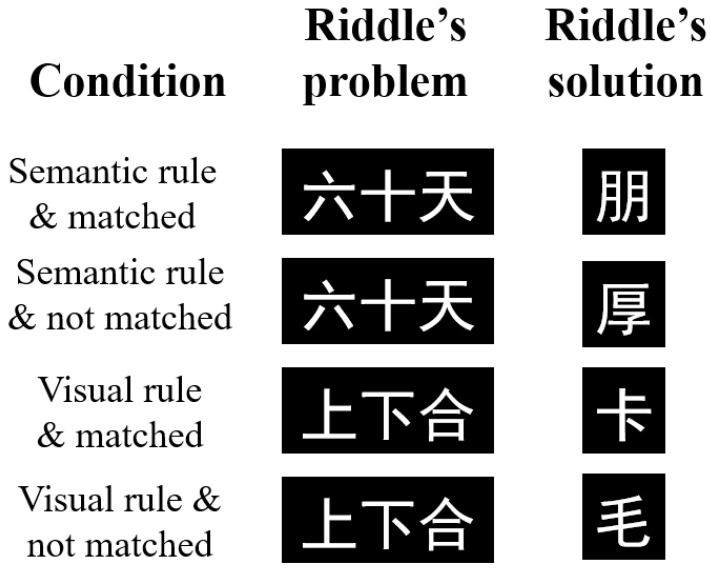
Examples of the materials in four conditions.

A major challenge in addressing this question is to manipulate perceptual and semantic insight within a paradigm, because traditional research confounds insight level with task type (visuo-perceptual insight is typically elicited by visual problems, whereas semantic insight is elicited by verbal problems). Chinese riddles, however, offer a solution. They typically consist of a puzzle stem (a problem) and a target solution, where the stem is a short phrase written in Chinese characters and the solution is one or more individual Chinese characters. Previous research has indicated that deciphering or understanding the solution of Chinese riddles can evoke moments of insight through different principles (e.g., Mai et al., 2004; Qiu et al., 2006; Y. Z. Wang, 2021; Zhao et al., 2014). Moreover, a Chinese character carries both visuo-perceptual information and semantic information (Luo et al., 2006; C. Y. Wu et al., 2012; Zhou & Marslen-Wilson, 1999). Correspondingly, the solution (a single character) can be reached by extracting the riddle’s underlying rule at either the visuo-perceptual level (i.e., visual-rule riddles) or the semantic level (i.e., semantic-rule riddles). Take the visual-rule riddles “上下合—卡” as an example. The key to solving the riddle’s problem “上下合” is to grasp the underlying visual rule: combining the graphical components “上” (above) and “下” (below) according to the character “合” (to combine). This allows the solver to derive the solution “卡”, in which “上” is positioned at the top and “下” at the bottom. By contrast, the key to unlocking the solution “朋” for the semantic-rule riddle stem “六十天” is to grasp the semantic rule: sixty days equate to two months. This connection holds because the literal meaning of “六十天” is sixty days, and the character “朋” is composed of two instances of “月 (month)” (see Figure 1 for examples). In short, solving visual-rule riddles relies primarily on visuo-perceptual processing, whereas solving semantic-rule riddles relies primarily on semantic processing (Y. Z. Wang, 2021).

In natural settings, self-generated insights arise unexpectedly (Metcalfe & Wiebe, 1987). By contrast, induced insights are more predictable and amenable to experimental manipulation, which makes them particularly valuable for learning and educational applications. Thus, we focus on comparing the memory effects of semantic versus perceptual induced insight in the present study. For this purpose, we introduced a Chinese riddle comprehension paradigm, in which participants were asked to judge whether they could comprehend the solution to each riddle (semantic-rule riddles or visual-rule riddles) after a brief problem-solving attempt during the learning stage. Then, they received an immediate recognition test (following a 2 min distraction task) or a delayed recognition test (after 24 h) and provided retrospective confidence judgments.

One aim of this study is to test the memory superiority effect for appropriateness-defined insight during solution comprehension for both semantic-rule and visual-rule riddles. To this end, we adopted the approach of prior work (Kizilirmak et al., 2016a, 2019, 2021; Zhang et al., 2025d) and included two additional control conditions with mismatched riddle–solution pairings, which produce incomprehension (non-insight). This paradigm is grounded in appropriateness-defined insight, which refers to a solution that is both consistent with prior knowledge and suitable for the problem context (Huang et al., 2018; Kizilirmak et al., 2016a, 2019; Paletz & Peng, 2008; Runco & Jaeger, 2012; Zhang et al., 2025d). Accordingly, the insight condition is defined as trials in which participants can grasp the logical relationship between matched solutions and problems (e.g., “卡” is the correct, matched solution to the visual-rule riddle “上下合”; likewise, “朋” is the correct, matched solution to the semantic-rule riddle “六十天”). In contrast, the non-insight condition is defined as trials in which participants cannot understand the connection between mismatched, inappropriate solutions and problems (e.g., “毛” is an incorrect, mismatched solution to “上下合”; similarly, “厚” is an incorrect, mismatched solution to “六十天”). These conditions served as counterpoints to the insightful (appropriate) comprehension conditions for both visual-rule and semantic-rule riddles, respectively (also see Figure 1 for examples).

Previous studies have indicated that the memory superiority effect of insight can be attributed to representational transformation via the integration of appropriate novel associations (e.g., S. Chen et al., 2021; Kizilirmak et al., 2019; Zhao et al., 2025). Accordingly, we hypothesize that, compared to the incomprehension/inappropriate condition (non-insight), insightful comprehension of the appropriate relationships between solutions and problems would lead to better recognition performance. Additionally, we aim to examine whether insight, particularly the integration of appropriate novel associations, benefits memory confidence judgment. Generally, individuals are more confident in their judgment for higher retrospective confidence judgment (RCJ, Chua et al., 2009; Dougherty et al., 2005; Hu et al., 2022; Martín-Luengo et al., 2021). Moreover, higher memory confidence judgment accuracy (MCJA) indicates better alignment between confidence ratings and actual memory performance (Conte et al., 2023; Fleming & Lau, 2014; Maniscalco & Lau, 2012). Previous research has shown that insight is associated with elevated RCJ (Ludmer et al., 2011; W. Shen et al., 2021; Zhang et al., 2025b), as well as superior MCJA (Zhang et al., 2025d). Based on these findings, we hypothesize that appropriate solutions (insight trials) will be associated with higher RCJ and MCJA than inappropriate solutions (non-insight trials).

The second and most important aim is to examine whether there are differences in the memory effects of induced semantic versus perceptual insight. First, previous studies have indicated that semantic insight and perceptual insight have distinct neurocognitive bases: while the former activates brain regions involved in semantic processing (e.g., anterior superior/middle temporal gyrus, Jung-Beeman et al., 2004; Tik et al., 2018), the latter activates visuo-perception-related regions (e.g., the visual and parietal cortices, Ludmer et al., 2011; Tang et al., 2016). Similarly, memory research indicates that semantic encoding activates semantic-specific brain regions (e.g., the middle and inferior frontal and parietal regions), which are different from those related to visuo-perceptual encoding (e.g., occipital regions) (West et al., 2026). Second, according to the levels-of-processing theory, compared with visuo-perceptual processing, semantic processing generates stronger and more durable memory traces, thereby leading to superior memory outcomes during the retrieval phase (Baddeley & Hitch, 2017; Craik & Tulving, 1975; Roediger et al., 2002; Craik & Rose, 2012). Consistently, several empirical studies have provided evidence that the semantic encoding condition yields better memory performance than the perceptual encoding condition (Friedman et al., 1996; Kuo et al., 2010; Nyhus & Curran, 2009; Paller et al., 1987). Third, comprehending semantic-rule riddles relies on representational transformation via the integration of semantic information, whereas comprehending visual-rule riddles depends on representational transformation via the integration of visuo-perceptual information. Accordingly, we hypothesize that comprehending the semantic rules of riddles will lead to better memory performance (higher recognition accuracy and faster recognition response times) than comprehending the visual rules of riddles. Meanwhile, we further predict that induced insight at the semantic level will elicit higher retrospective confidence judgments (RCJ) and greater memory confidence judgment accuracy (MCJA) than induced insight at the perceptual level.

The third aim is to examine the role of the “aha!” experience in the relationship between insight and memory. Specifically, previous studies have indicated that insight (vs. non-insight) elicits stronger “aha!” experiences (Bowden & Jung-Beeman, 2007; Zhang et al., 2025d). We aim to replicate this effect, and we therefore hypothesize that comprehending the logical relationship in matched problem–solution pairs (insight) will elicit higher “aha!” ratings than being unable to grasp the connection in mismatched, inappropriate (non-insight) pairs. Furthermore, we aim to test whether “aha!” experiences differ across levels of insight. It is hypothesized that comprehending semantic-rule riddles elicits stronger “aha!” experiences than comprehending visual-rule riddles.

Moreover, evidence suggests that “aha!” feelings contribute to the memory superiority effect of insight. For example, Danek and colleagues, using magic tricks, found that stronger “aha!” feelings predicted higher retention rates for magic solutions after 1 or 2 weeks (Danek et al., 2013; Danek & Wiley, 2020). Additionally, Salvi et al. (2024) found that individuals had better memory for incidental scholastic facts that were encountered in close temporal proximity to spontaneous or induced “aha!” moments (compared to no “aha!”). Moreover, Ludmer et al. (2011) revealed that activation levels in the amygdala, a region closely linked to emotional processing, significantly predicted accurate recall of insight events one week after problem-solving. Similarly, Kizilirmak and colleagues used RATs to observe activation in the left amygdala and left striatum during the encoding phase, which predicted remembered (vs. forgotten) triad solutions 24 h later (Kizilirmak et al., 2016a). Accordingly, we hypothesize that higher “aha!” ratings would predict better memory performance (e.g., higher hit rates, shorter response times).

In short, the primary aim of this study is to examine the memory superiority effect of induced insight at both the semantic and perceptual levels, and to compare the memory effects of semantic versus perceptual insight by employing a riddle learning–testing paradigm. In the learning phase, participants judged whether they understood the relationship between each riddle’s solution and its corresponding problem. They completed tasks across four conditions: comprehending the visual or semantic rules of riddles in which the solution matched the riddle problem (the matched, appropriate/insight conditions), or in which the solution mismatched the problem (the mismatched, inappropriate/non-insight conditions). In the immediate (2 min post-distraction) and delayed (24 h post-learning) testing phases, participants were asked to judge whether each solution (a Chinese character) had been presented before (old) or was new. We measured multiple indices of memory performance (including hit rates, recognition accuracy, and recognition response times) and memory confidence judgments (including retrospective confidence judgments and confidence judgment accuracy). These measures allowed us to assess the effects of comprehension in the matched/appropriate condition (vs. incomprehension in the mismatched/inappropriate condition), as well as comprehending semantic rules (vs. visual rules).

## 2. Materials and Methods

### 2.1. Participants

According to the sample size estimation based on MorePower 6.0.4 software (Campbell & Thompson, 2012), a minimum of 28 participants were needed, with an effect size η^2^ = 0.24 (X. Wu et al., 2018), power (1 − β) = 0.80, α err prob = 0.05 and MSE = 1. Although the a priori power analysis indicated that *N* = 28 would be sufficient to detect the key effect of interest: the three-way interaction of 2 (Riddle type: semantic-rule vs. visual-rule) × 2 (Match type: matched vs. unmatched) × 2 (Recognition time: immediate vs. delayed), we collected data from 40 participants to account for potential exclusions and to ensure adequate power for planned secondary analyses. Forty university students voluntarily participated in this study. Data from five participants were excluded: two participants had excessively high solution rates (solution rate 1 = 0.89, solution rate 2 = 0.58) during the 3 s riddle-guessing phase (see Section 2.4 for rationale). One participant had a high comprehension rate for the riddles for which the problem did not match the solution (comprehension rate = 0.86) in the comprehension phase, and one participant had a low comprehension rate of the matched materials (comprehension rate = 0.27), and another participant had a hit rate of zero in the immediate test for the riddles in the visual-rule & unmatched condition. Ultimately, the valid data of 35 participants (23 females; age: 20.09 ± 1.13) were included. All participants were right-handed, native Chinese speakers (using simplified Chinese characters), had normal or corrected-to-normal vision, reported no physical discomfort, and had not participated in any similar experiment previously. This study received ethical approval from the local institutional review board.

### 2.2. Materials

The Chinese character riddles used in this study were drawn from the Internet and from two books, *The New Riddles of Chinese Characters Compendium* (J. Shen, 2006) and *The Grand Dictionary of Chinese Character Riddles* (Sun, 2002), and were selected according to the definitions of semantic-rule and the visual-rule riddles provided by Y. Z. Wang (2021). For semantic-rule riddles, solving the riddle involves replacing key characters or phrases in the problem with semantically synonymous components that compose the solution. For visual-rule riddles, solving requires the visuospatial manipulation of the structural elements of the riddle’s problem. Two types of riddle materials were initially selected in accordance with the following criteria: (1) all Chinese characters used in the problems and solutions appear in the *Modern Chinese Frequency Dictionary* (H. Wang, 1986), ensuring that they are all common and contain no obscure characters; (2) the riddle problem is easy to understand and requires no specialized knowledge to solve; and (3) each riddle consist of a problem composed of three Chinese characters, and the solution is a single, unique Chinese character.

Based on these criteria, a total of 304 three-character riddles were initially selected, with 152 semantic-rule riddles and 152 visual-rule riddles. For example, in the semantic-rule riddle “六十天—朋”, the riddle stem (a problem) “六十天” literally means sixty days. Because the Chinese character “朋” is composed of two “月” (month) components, and one month is approximately 30 days, two months together amount to 60 days, yielding the solution “朋”. In contrast, in the visual-rule riddle “上下合—卡”, the character “合” denotes the visuospatial combination of “上” (above) and “下” (below), forming the solution “卡.” Additionally, to create the unmatched (control) conditions, each riddle stem was randomly paired with a solution from a different riddle. For example, the stem “六十天” bears no semantic or visual relation to “厚”, forming a mismatched pair. Thus, the unmatched pair “六十天—厚” serves as the control for the matched pair “六十天—朋.” Similarly, “上下合” and “毛” are unrelated, so the unmatched pair “上下合—毛” acted as the control for the matched pair “上下合—卡.” (See Figure 1 for examples.)

Twenty-one university students were recruited to rate the three-character riddle stems (e.g., “六十天”) on a five-point scale for semantic conventionality, familiarity, concreteness, and imagery. Semantic conventionality refers to the degree to which the phrase aligns with everyday linguistic usage (Y. Z. Wang, 2021). The remaining three constructs were defined as follows (Altarriba et al., 1999; Gilhooly & Logie, 1980): Familiarity refers to how often a phrase or word is encountered or used in daily life. Concreteness refers to the degree to which a phrase or word denotes a tangible, physical entity. Imagery refers to the ease with which a person can form a mental image of a given phrase or word. An independent group of 20 university students rated the riddles (e.g., “六十天—朋”) on a five-point scale for “aha!” experience, comprehensibility, ingenuity/novelty, appropriateness, and difficulty. All subjective ratings were collected using a 5-point Likert scale (1 = not at all, 5 = very much). After excluding riddles with scores below 3 on any of the key indices (semantic conventionality, ingenuity/novelty, and appropriateness), 228 riddle pairs remained, including 114 semantic-rule riddle pairs and 114 visual-rule riddle pairs. From these, 120 riddle pairs (60 semantic-rule and 60 visual-rule pairs) were selected as the formal experimental materials. Results of independent-samples *t*-tests conducted on all measured variables for semantic-rule and visual-rule riddles are presented in Table 1.

For the recognition test, the 120 riddle pairs were further equally divided into matched and unmatched conditions, with 30 pairs per condition (i.e., 30 pairs of matched or unmatched riddles for each semantic-rule or visual-rule type). To avoid the influence of testing effects (Wong & Lim, 2022), 50% of the items were assigned to the immediate recognition phase, and 50% to the delayed phase. Accordingly, participants were presented with 60 old riddles (15 pairs of semantic-rule and matched riddles, 15 pairs of semantic-rule and unmatched riddles, 15 pairs of visual-rule and matched riddles, and 15 pairs of visual-rule and unmatched riddles) and 60 new characters during each recognition test (immediate or delayed). The new characters were sourced from the Chinese character frequency list of the Modern Chinese Corpus, which contains 20 million characters and is available online at https://lingua.mtsu.edu/chinese-computing/statistics/char/list.php?Which=TO (accessed on 14 July 2023). There was no significant difference in character frequency between old and new characters (*t* (238) = 0.01, *p* = 1.000). (It should be noted that we employed a learning–testing paradigm in the present study. Accordingly, we consistently refer to stimuli as old or new during the test/recognition phase. Specifically, old stimuli (riddles or characters) are those previously presented during the learning phase, while new stimuli (riddles or characters) are those never presented during the learning phase.)

### 2.3. Procedure

**Figure 2:**
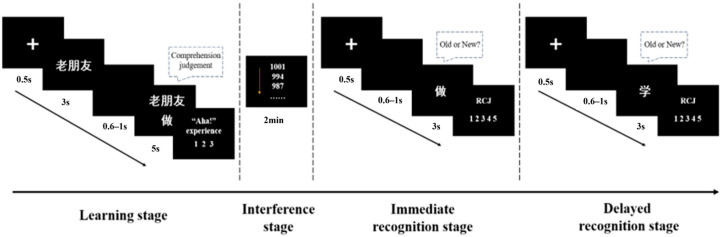
Examples of the procedure. (RCJ: retrospective confidence judgment. “老朋友” means old friend; “做” means do, consisting of “**亻**” which is equals to “人” meaning people and “故” which is an adjective meaning “from the past”; “学” means learning).

The experimental procedures and materials were presented using E-Prime 2.0 software. This experiment employed a learning–testing paradigm and was divided into four stages (see Figure 2). In the learning phase, participants were first given 3 s to solve the presented riddle stem (e.g., “六十天”). They pressed the Enter key only if they generated a solution within 3 s; otherwise, they made no response and they were not required to report or record any solution they generated. Subsequently, the riddle stem was displayed along with a solution for 5 s; the solution was either matched (e.g., “朋”) or mismatched (e.g., “厚”) with the stem (e.g., “六十天”). Participants were instructed to press the “F” key if they comprehended the relationship between the stem and solution, or the “J” key if they did not. Following this, participants rated their “aha!” experiences on a 3-point scale (1 = no “aha!”, 2 = neutral, 3 = clear “aha!”). “Aha!” experiences were defined as follows: “Once you comprehend the solution to a problem you were previously unable to solve, you feel a sudden, surprising flash of clarity, as if the answer dawns on you instantly; at that moment, you likely experience the enlightening sensation of ‘Aha! That’s it!’—a classic ‘penny drop’ moment” (Bowden & Jung-Beeman, 2007; Salvi et al., 2016). In the interference phase, participants completed a 2 min distraction task in which they serially subtracted 7 from 1001 using paper and pencil. In the immediate and delayed (1-day) recognition phases, participants were instructed to judge quickly and accurately whether the presented Chinese characters were old (previously shown in the learning phase) or new (never shown in the learning phase) within a 3 s response window. They pressed the “F” key for old characters and the “J” key for new characters. After each recognition judgment, participants rated their retrospective confidence judgment (RCJ) on a 5-point scale: a score of 1 indicated complete uncertainty in their recognition decision, while a score of 5 indicated maximal confidence. During the formal experiment, participants were not fully informed of the study’s true purpose; they were only told they would complete a riddle judgment task. Additionally, they were informed that they would return to completing a second task at the same time the following day. This procedure prevented participants from using intentional memorization strategies. Key press assignments, riddle matching conditions, and daily testing schedules (Day 1 vs. Day 2) were fully counterbalanced across participants. Before the formal experiment, participants completed 12 practice riddle pairs to familiarize themselves with the task. Each participant completed a total of 120 learning trials and 240 test trials. The entire experimental session, including instructions and practice, lasted approximately 35–45 min.

All trials of semantic-rule and visual-rule riddles were intermixed and presented in a randomized order during both the learning and recognition phases. Participants were allowed a self-paced break after every 33 riddle pairs to reduce fatigue. They were not informed of the distinction between visual-rule and semantic-rule riddles at any stage of the experiment, nor were they given any hints regarding the perceptual or semantic processing principles underlying the riddle solutions.

### 2.4. Data Collection and Analysis

The experimental data were collected using E-Prime 2.0 and analyzed with SPSS 27.0.

During the learning phase, the following metrics were recorded and computed for each experimental condition: (1) Solution rate was defined as the proportion of trials in which participants generated a solution (pressed Enter) during the 3 s riddle-guessing window, relative to the total number of trials per condition. Consistent with prior research (e.g., Kizilirmak et al., 2016a; Ludmer et al., 2011; Zhang et al., 2025d), trials solved within this 3 s window were classified as spontaneously solved and subsequently excluded from further analyses, because the present study focused on the memory effects of induced insight. Furthermore, we applied an exclusion criterion for participants: any participant with an average solution rate exceeding 0.5 was excluded from all subsequent analyses. This is because participants normally have a very low probability of solving the riddle stems (i.e., identifying the correct solution) within such a brief timeframe (3 s). Thus, elevated solution rates likely reflect inadequate task engagement rather than genuine problem-solving. Furthermore, this study centers on the memory effects of induced insight tied to comprehension trials for riddles not solved in the initial 3 s window. High solution rates produce outliers that drastically reduce the number of valid trials available for subsequent comprehension and recognition analyses. After excluding spontaneously solved trials, the following indices were computed: (2) Comprehension rate was calculated as the proportion of trials in which participants pressed the comprehension key within each condition; (3) Comprehension judgment response time was computed as the average latency for accurate responses (i.e., pressing “F” for matched riddles and “J” for unmatched riddles). Because raw response times exhibited positive skewness and violated the normality assumption required for parametric tests, a natural log transformation was applied to normalize the distribution and minimize the impact of extreme outliers; (4) “Aha!” experience ratings were calculated as the mean score for correct comprehension trials.

During the recognition phase, trials were first excluded if they had been solved within 3 s during the learning phase, if participants made incorrect responses during the comprehension judgment (specifically, pressing the non-comprehension key in match conditions or the comprehension key in non-match conditions), or if no response was provided during comprehension or recognition judgments. Next, memory performance was computed using the following indices: hit rate, recognition accuracy, and recognition response time. Memory confidence judgments were assessed using retrospective confidence judgments (RCJ) and memory confidence judgment accuracy: (5) Hit rate: defined as the number of correctly recognized old characters divided by the total number of old-character trials; (6) Recognition rate: calculated as the difference between the rate of old characters correctly judged as “old” and the rate of new characters incorrectly judged as “old”, formulated as recognition rate = hit rate (old) − false-alarm rate (new); (7) Recognition response time (RT): representing the average response time for correctly recognized old characters. Natural log transformation was applied here for the same reasons stated above; (8) Retrospective confidence judgment (RCJ): reflecting the mean RCJ score for correctly recognized old characters; and (9) Memory confidence judgment accuracy: measured by the area under the Type 2 receiver operating characteristic (ROC) curve (relative to the diagonal line) constructed from objective task performance and subjective RCJ scores during the recognition phase (Fleming & Lau, 2014). To construct Type 2 ROC curves, confidence thresholds were divided dynamically to generate multiple data points. Calculation of memory confidence judgment accuracy was based on the Type 2 ROC MATLAB R2018a code provided by Fleming and Lau (2014) (http://www.frontiersin.org/journal/10.3389/fnhum.2014.00443/abstract, accessed on 16 October 2024).

To address the core research question regarding participants’ memory confidence judgment, we employed a rigorous approach for assessing memory confidence judgment accuracy. Given that traditional correlation measures (e.g., gamma coefficient) are susceptible to contamination by confidence judgment bias—a participant with a conservative bias (a general reluctance to use high confidence ratings) will yield a different gamma value than a participant with a liberal bias, even if their underlying ability to monitor their own performance is identical. In contrast, the Type 2 ROC analysis circumvents this issue. Specifically, memory confidence judgment accuracy was assessed using a Type 2 Receiver Operating Characteristic (ROC) analysis (Fleming & Lau, 2014). This non-parametric approach was selected for its critical advantage of providing a bias-free measure of a participant’s ability to discriminate between their own correct and incorrect judgments, independent of their overall tendency to use the confidence scale (i.e., confidence judgment bias). It is constructed by treating each level of the confidence rating scale as a criterion for classifying a trial as “high” vs. “low” confidence. The analysis proceeds as follows: First, for a given confidence level (e.g., rating 5 on a 5-point scale), we calculate the Type 2 hit rate (Type 2 hit rate (H2), defined as the proportion of correct trials assigned a confidence rating ≥5) and the Type 2 false alarm rate (Type 2 false alarm rate (FA2), defined as the proportion of incorrect trials assigned a confidence rating ≥5). This provides one point on the ROC plot. This process is iterated for all possible confidence criteria (e.g., ratings ≥3, ≥2, etc.), generating a series of (FA2, H2) points. The resulting Type 2 ROC curve is plotted H2 against FA2 for all criteria. The area under this Type 2 ROC curve (AUROC2) served as our measure of confidence judgment sensitivity. AUROC2 of 0.5 indicates chance-level discrimination (no ability to distinguish correct from incorrect trials), whereas values significantly above 0.5 indicate above-chance sensitivity. The major strength of this measure is that it is derived from the full distribution of confidence ratings across criteria and is not influenced by an individual’s arbitrary placement of a single criterion, thereby yielding a less biased index of confidence judgment accuracy (Galvin et al., 2003; Fleming & Lau, 2014). This method is particularly suited for our design, which employed a multi-point confidence scale and allowed us to construct a complete ROC function for each participant.

## 3. Results

### 3.1. Learning Stage

A total of 4800 trials were included in the analysis of solution rates, comprising 586 trials in which a solution was generated within 3 s and 4214 trials where answers were not generated within 3 s. The trials in which no answer was generated within 3 s were included in the subsequent analyses of comprehension rate, response time for comprehension judgment and “aha!” experiences. The descriptive statistics for each variable across conditions of the learning phase are presented in Table 2. Separate two-way repeated-measures ANOVAs were conducted on each variable, with Riddle type (semantic-rule vs. visual-rule) and Match type (matched vs. unmatched).

#### 3.1.1. Solution Rate

A 2 (riddle type: semantic-rule vs. visual-rule) × 2 (match type: matched vs. unmatched) repeated-measures ANOVA was conducted on solution rate. There was a significant main effect of riddle type, indicating that the semantic-rule riddles (*M* = 0.06, *SE* = 0.02, 95% CI = [0.03, 0.09]) had a lower solution rate compared to visual-rule riddles (*M* = 0.12, *SE* = 0.02, 95% CI = [0.07, 0.16]), *F* (1, 34) = 29.71, *p* < 0.001, η*_p_*^2^ = 0.47. The main effect of Match type [*F* (1, 34) = 1.76, *p* = 0.193], and the interaction between Riddle type and Match type were not significant [*F* (1, 34) = 0.00, *p* = 1.000].

#### 3.1.2. Comprehension Rate

The same two-way repeated-measures ANOVA was conducted on comprehension rate. There was a significant main effect of Riddle type, indicating that the semantic-rule riddles (*M* = 0.38, *SE* = 0.02, 95% CI = [0.34, 0.43]) had lower comprehension rates compared to the visual-rule riddles (*M* = 0.44, *SE* = 0.02, 95% CI = [0.41, 0.47]), *F* (1, 34) = 11.67, *p* = 0.002, η*_p_*^2^ = 0.26. There was also a significant main effect of Match type, indicating that the matched trials (*M* = 0.71, *SE* = 0.03, 95% CI = [0.65, 0.76]) had higher comprehension rates compared to that of the unmatched trials (*M* = 0.12, *SE* = 0.02, 95% CI = [0.08, 0.15]), *F* (1, 34) = 536.11, *p* < 0.001, η*_p_*^2^ = 0.94. The interaction between Riddle type and Match type was significant, *F* (1, 34) = 11.59, *p* = 0.002, η*_p_*^2^ = 0.25.

Further simple effect analyses revealed that the comprehension rate was significantly higher in the matched condition than in the unmatched condition for the semantic-rule riddle, *F* (1, 34) = 263.71, *p* < 0.001, η*_p_*^2^ = 0.89, 95% CI for the difference = [0.46, 0.59], and also significantly higher in the matched condition (*M* = 0.76, *SE* = 0.02, 95% CI = [0.71, 0.81]) than in the unmatched condition for the visual-rule riddle, *F* (1, 34) = 480.84, *p* < 0.001, η*_p_*^2^ = 0.93, 95% CI for the difference = [0.59, 0.71]. In addition, the comprehension rate was significantly lower for the semantic-rule riddle than for the visual-rule riddle in the matched condition, *F* (1, 34) = 14.64, *p* < 0.001, η*_p_*^2^ = 0.30, 95% CI for the difference = [−0.18, −0.06]. However, there was no significant difference between the semantic-rule riddle and the visual-rule riddle in the unmatched condition, *F* (1, 34) = 0.05, *p* = 0.826, η*_p_*^2^ = 0.001.

#### 3.1.3. Response Time for Comprehension Judgment

The same two-way repeated-measures ANOVA was conducted on Response time for comprehension judgment. There was a significant main effect of Riddle type, indicating that the semantic-rule riddles (*M* = 2748.30, *SE* = 76.37, 95% CI = [2593.12, 2903.49]) were associated with longer response times than the visual-rule riddles (*M* = 2599.82, *SE* = 74.56, 95% CI = [2448.30, 2751.34]), *F* (1, 34) = 16.80, *p* < 0.001, η*_p_*^2^ = 0.33. There was also a significant main effect of Match type, indicating that responses to the matched riddles (*M* = 2587.22, *SE* = 71.79, 95% CI = [2441.32, 2733.12]) required shorter response times than responses to the unmatched riddles (*M* = 2760.90, *SE* = 88.19, 95% CI = [2581.69, 2940.12]), *F* (1, 34) = 6.87, *p* = 0.013, η*_p_*^2^ = 0.17. The interaction between Riddle type and Match type was significant, *F* (1, 34) = 22.67, *p* < 0.001, η*_p_*^2^ = 0.40.

Further simple effect analyses revealed that response time for comprehension judgment was significantly shorter in the matched condition than in the unmatched condition for the visual-rule riddle, *F* (1, 34) = 17.07, *p* < 0.001, η*_p_*^2^ = 0.33, 95% CI for the difference = [−494.49, −168.43], but there was no significant difference for the semantic-rule riddle, *F* (1, 34) = 0.06, *p* = 0.815. In addition, the response time of comprehension judgment was significantly longer for the semantic-rule riddle than for the visual-rule riddle in the matched condition, *F* (1,34) = 30.14, *p* < 0.001, η*_p_*^2^ = 0.47, 95% CI for the difference = [192.89, 419.63], but there was no significant difference between them in the unmatched condition, *F* (1, 34) = 0.05, *p* = 0.820.

#### 3.1.4. “Aha!” Experiences

The same two-way repeated-measures ANOVA was conducted on “aha!” experiences. There was a significant main effect of Riddle type, indicating that the semantic-rule riddles (*M* = 1.96, *SE* = 0.02, 95% CI = [1.91, 2.00]) had stronger “aha!” experiences than the visual-rule riddles (*M* = 1.90, *SE* = 0.03, 95% CI = [1.84, 1.97]), *F* (1, 34) = 4.33, *p* = 0.045, η*_p_*^2^ = 0.11. There was also a significant main effect of Match type, indicating that participants reported stronger “aha!” experiences in the matched condition (*M* = 2.74, *SE* = 0.04, 95% CI = [2.67, 2.82]) than the unmatched condition (*M* = 1.12, *SE* = 0.03, 95% CI = [1.06, 1.17]), *F* (1, 34) = 1533.18, *p* < 0.001, η*_p_*^2^ = 0.98. The interaction between Riddle type and Match type was not significant, *F* (1, 34) = 1.64, *p* = 0.209.

### 3.2. Recognition Stage

The descriptive statistics of hit rate, recognition rate, recognition response time, retrospective confidence judgment, and memory confidence judgment accuracy for the eight conditions in the recognition stage are shown in Table 3. A series of 2 (riddle type: semantic-rule vs. visual-rule) × 2 (match type: matched vs. unmatched) × 2 (recognition time: immediate vs. delayed) repeated-measures ANOVA was conducted, one for each dependent variable.

#### 3.2.1. Hit Rate

The three-way repeated-measures ANOVA on hit rate showed that there was a significant main effect of Riddle type, indicating that the semantic-rule riddles (*M* = 0.85, *SE* = 0.02, 95% CI = [0.81, 0.89]) had a higher hit rate than the visual-rule riddles (*M* = 0.76, *SE* = 0.02, 95% CI = [0.71, 0.80]), *F* (1, 34) = 48.71, *p* < 0.001, η*_p_*^2^ = 0.589, power = 1.000. There was also a significant main effect of Match type, indicating that the matched trials (*M* = 0.84, *SE* = 0.02, 95% CI = [0.80, 0.88]) had a higher hit rate than the unmatched trials (*M* = 0.76, *SE* = 0.03, 95% CI = [0.71, 0.82]), *F* (1, 34) = 19.68, *p* < 0.001, η*_p_*^2^ = 0.367, power = 0.991. There was also a significant main effect of Recognition time, indicating that the immediate recognition (*M* = 0.85, *SE* = 0.02, 95% CI = [0.81, 0.89]) had a higher hit rate than the delayed recognition (*M* = 0.76, *SE* = 0.03, 95% CI = [0.71, 0.81]), *F* (1, 34) = 22.37, *p* < 0.001, η*_p_*^2^ = 0.397, power = 0.996. All interactions were not significant: Riddle type × Match type × Recognition time *F* (1, 34) = 0.06, *p* = 0.811, η*_p_*^2^ = 0.002, power = 0.057; Riddle type × Match type *F* (1, 34) = 0.59, *p* = 0.448, η*_p_*^2^ = 0.017, power = 0.116; Riddle type × Recognition time *F* (1, 34) = 1.11, *p* = 0.299, η*_p_*^2^ = 0.032, power = 0.178; and Match type × Recognition time *F* (1, 34) = 1.92, *p* = 0.175, η*_p_*^2^ = 0.053, power = 0.268.

#### 3.2.2. Recognition Rate

The three-way repeated-measures ANOVA on recognition rate showed that there was a significant main effect of Riddle type, indicating that the semantic-rule riddles (*M* = 0.60, *SE* = 0.02, 95% CI = [0.55, 0.65]) had a higher recognition rate than that of the visual-rule riddles (*M* = 0.51, *SE* = 0.03, 95% CI = [0.44, 0.57]), *F* (1, 34) = 48.71, *p* < 0.001, η*_p_*^2^ = 0.589, power = 1.000. There was also a significant main effect of Match type, indicating that the matched trials (*M* = 0.60, *SE* = 0.03, 95% CI = [0.54, 0.64]) had a higher recognition rate than that of the unmatched trials (*M* = 0.51, *SE* = 0.03, 95% CI = [0.45, 0.57]), *F* (1, 34) = 19.68, *p* < 0.001, η*_p_*^2^ = 0.367, power = 0.991. There was also a significant main effect of Recognition time, indicating that the immediate recognition condition (*M* = 0.67, *SE* = 0.03, 95% CI = [0.61, 0.72]) had a higher rate than the delayed recognition condition (*M* = 0.44, *SE* = 0.03, 95% CI = [0.37, 0.50]), *F* (1, 34) = 97.84, *p* < 0.001, η*_p_*^2^ = 0.742, power = 1.000. All interactions were not significant: Riddle type × Match type × Recognition time, *F* (1, 34) = 0.058, *p* = 0.811, η*_p_*^2^ = 0.002, power = 0.057; Riddle type × Match type, *F* (1, 34) = 0.59, *p* = 0.448, η*_p_*^2^ = 0.017, power = 0.116; Riddle type × Recognition time, *F* (1, 34) = 1.11, *p* = 0.299, η*_p_*^2^ = 0.032, power = 0.178; Match type × Recognition time, *F* (1, 34) = 1.92, *p* = 0.175, η*_p_*^2^ = 0.053, power = 0.268.

#### 3.2.3. RT for Recognition

After applying a logarithmic transformation to the reaction times, the three-way repeated-measures ANOVA on RT for recognition showed that there was a significant main effect of Match type, indicating that the matched trials (*M* = 3.03, *SE* = 0.16, 95% CI = [3.00, 3.06]) required shorter RT than that of not matched trials (*M* = 3.07, *SE* = 0.16, 95% CI = [3.04, 3.11]), *F* (1, 34) = 39.18, *p* < 0.001, η*_p_*^2^ = 0.535, power = 1.000. There was a significant main effect of recognition time, indicating that immediate recognition condition (*M* = 3.04, *SE* = 0.15, 95% CI = [3.00, 3.07]) required shorter time than delayed recognition condition (*M* = 3.07, *SE* = 0.19, 95% CI = [3.03, 3.11]), *F* (1, 34) = 6.18, *p* = 0.018, η*_p_*^2^ = 0.154, power = 0.676. However, the main effect of Riddle type was not significant, *F* (1, 34) = 0.50, *p* = 0.483, η*_p_*^2^ = 0.015, power = 0.108. All interactions were not significant: Riddle type × Match type × and Recognition time, *F* (1, 34) = 1.40, *p* = 0.246, η*_p_*^2^ = 0.039, power = 0.207; Riddle type × Match type, *F* (1, 34) = 1.10, *p* = 0.302, η*_p_*^2^ = 0.031, power = 0.173; Riddle type × Recognition time, *F* (1, 34) = 0.17, *p* = 0.685, η*_p_*^2^ = 0.005, power = 0.069; Match type × Recognition time, *F* (1, 34) = 0.06, *p* = 0.812, η*_p_*^2^ = 0.002, power = 0.057.

#### 3.2.4. Retrospective Confidence Judgment

The three-way repeated-measures ANOVA on retrospective confidence judgment showed that there was a significant main effect of Riddle type, indicating that the semantic-rule riddles (*M* = 4.55, *SE* = 0.05, 95% CI = [4.44, 4.66]) had higher RCJ than the visual-rule riddles (*M* = 4.43, *SE* = 0.07, 95% CI = [4.29, 4.56]), *F* (1, 34) = 17.82, *p* < 0.001, η*_p_*^2^ = 0.344, power = 0.984. There was also a significant main effect of Match type, indicating that the matched trials (*M* = 4.60, *SE* = 0.05, 95% CI = [4.49, 4.71]) had higher RCJ than that of the unmatched trials (*M* = 4.38, *SE* = 0.07, 95% CI = [4.24, 4.51]), *F* (1, 34) = 48.54, *p* < 0.001, η*_p_*^2^ = 0.588, power = 1.000. There was also a significant main effect of Recognition time, indicating that the immediate recognition condition (*M* = 4.63, *SE* = 0.05, 95% CI = [4.52, 4.74]) had higher RCJ than the delayed recognition condition (*M* = 4.35, *SE* = 0.08, 95% CI = [4.19, 4.51]), *F* (1, 34) = 20.83, *p* < 0.001, η*_p_*^2^ = 0.380, power = 0.993. The three-way interaction was not significant, *F* (1, 34) = 0.17, *p* = 0.684, η*_p_*^2^ = 0.005, power = 0.069. However, the two-way interaction between riddle type and match type was significant, *F* (1, 34) = 8.59, *p* = 0.006, η*_p_*^2^ = 0.202, power = 0.813. The other two-way interactions were not significant: riddle type × recognition time, *F* (1, 34) = 1.12, *p* = 0.297, η*_p_*^2^ = 0.032, power = 0.178; match type × recognition time, *F* (1, 34) = 0.86, *p* = 0.362, η*_p_*^2^ = 0.025, power = 0.148.

The interaction between Riddle type and Match type was significant, *F* (1, 34) = 8.59, *p* = 0.006, η*_p_*^2^ = 0.20. Further simple effect analyses revealed that RCJ was significantly higher in the matched condition than in the unmatched condition for the semantic-rule riddle, *F* (1, 34) = 40.36, *p* < 0.001, η*_p_*^2^ = 0.543, power = 1.000, 95% CI for the difference = [0.21, 0.41], and for the visual-rule riddle, *F* (1, 34) = 13.62, *p* < 0.001, η*_p_*^2^ = 0.286, power = 0.947, 95% CI for the difference = [0.06, 0.22]. In addition, RCJ was also significantly higher for the semantic-rule riddle than for the visual-rule riddle in the matched condition, *F* (1, 34) = 17.91, *p* < 0.001, η*_p_*^2^ = 0.345, power = 0.983, 95% CI for the difference = [0.11, 0.32], but there was no significant difference between the two in the unmatched condition, *F* (1, 34) = 1.55, *p* = 0.221, η*_p_*^2^ = 0.044, power = 0.220. Moreover, we computed the difference in RCJ between matched and unmatched trials separately for semantic-rule and visual-rule riddles for each participant, then conducted a paired-samples *t*-test on these two difference scores. The results showed that the RCJ difference was significantly larger for semantic-rule riddles (*M* = 0.30, *SE* = 0.05) than for visual-rule riddles (*M* = 0.13, *SE* = 0.04), *t* (34) = 2.71, *p* = 0.011, Cohen’s *d* = 0.46, power = 1.000.

#### 3.2.5. Memory Confidence Judgment Accuracy

The three-way repeated-measures ANOVA on memory confidence judgment accuracy showed that there was a significant main effect of Riddle type, indicating that the semantic-rule riddles (*M* = 0.72, *SE* = 0.01, 95% CI = [0.70, 0.74]) had higher accuracy than that of the visual-rule riddles (*M* = 0.69, *SE* = 0.01, 95% CI = [0.66, 0.72]), *F* (1, 34) = 9.98, *p* = 0.003, η*_p_*^2^ = 0.227, power = 0.866. There was also a significant main effect of Recognition time, indicating that the immediate recognition condition (*M* = 0.72, *SE* = 0.01, 95% CI = [0.70, 0.75]) had higher accuracy than that of the delayed recognition condition (*M* = 0.68, *SE* = 0.02, 95% CI = [0.65, 0.71]), *F* (1, 34) = 7.04, *p* = 0.012, η*_p_*^2^ = 0.172, power = 0.733. There was a trend toward a main effect of Match type, *F* (1, 34) = 3.05, *p* = 0.090, η*_p_*^2^ = 0.082, power = 0.395. All interactions were not significant: Riddle type × Match type, and Recognition time *F* (1, 34) = 0.10, *p* = 0.759, η*_p_*^2^ = 0.003, power = 0.061; Riddle type × Match type *F* (1, 34) = 0.66, *p* = 0.424, η*_p_*^2^ = 0.019, power = 0.124; Riddle type × Recognition time *F* (1, 34) = 0.05, *p* = 0.822, η*_p_*^2^ = 0.002, power = 0.057; and Match type × Recognition time *F* (1, 34) = 0.07, *p* = 0.793, η*_p_*^2^ = 0.002, power = 0.057.

#### 3.2.6. Relations Between “Aha!” Experiences and Memory Performance

In order to examine the potential role played by “aha!” experiences in memory performance, Pearson correlations between “aha!” experiences and the memory indicators were computed in the matched condition, as shown in Table 4. The results indicated that “aha!“ experiences were not significantly correlated with hit rate, recognition rate, recognition reaction time, RCJ, memory confidence judgment accuracy.

#### 3.2.7. Liner-Mixed-Model Analyses Controlling for Material Effects

Considering that there are significant differences in “aha” experience, comprehensibility, ingenuity/novelty, appropriateness, and difficulty ratings between semantic-rule riddles and visual-rule riddles, we conducted additional linear mixed-effects model analyses. The results indicate that there were significant effects of riddle type on memory performance after controlling for inherent material differences via random intercepts for items. The detailed analysis and results are as follows.

In this experimental study, hit rate was conceptualized as a dichotomous variable and analyzed using a Generalized Linear Mixed Model (GLMM). Response time, by contrast, was treated as a continuous variable and analyzed using a Linear Mixed Model (LMM). Given that the distribution of response times displayed a notable skewness, a logarithmic transformation was implemented to approximate a normal distribution. Additionally, the retrospective confidence judgment (RCJ) was classified as an ordinal variable and analyzed utilizing an ordinal logistic regression model.

Because these three models are fitted at the trial level, they afford greater statistical power. Recognition rate is calculated based on hit rate and false alarm rate, whereas memory confidence judgment accuracy is indirectly derived from RCJ and hit rate. Consequently, trial-level analysis is not feasible for these metrics. Accordingly, subsequent analyses focus exclusively on hit rate, recognition RT, and RCJ.

Although we were unable to collect subjective ratings for the unmatched trials, “aha!” experience, difficulty, and other relevant variables are inherent to the materials themselves. By incorporating “Materials” as a random intercept in the model, we effectively estimate a unique baseline value for each stimulus item. This approach rigorously controls for variance in the dependent variable attributable to time-invariant, intrinsic material characteristics. This method is widely recognized as a robust technique for accounting for between-item differences, as it enables us to partition variance stemming from material variability out of the error term, thus producing a cleaner and more precise estimate of the fixed effects.

The analysis was specified with riddle type, recognition time, and match type as fixed factors, and participant and material as random factors. If the maximal random-effects model failed to converge, the model was simplified by sequentially removing random terms. First, subject-related random slopes were eliminated, followed by any remaining subject-specific variance components, until the model achieved successful convergence.

Hit rate was analyzed using a GLMM. Results revealed a significant main effect of Riddle type, indicating that semantic-rule riddles (*M* = 0.83, *SE* = 0.01) yielded a higher hit rate than visual-rule riddles (*M* = 0.74, *SE* = 0.01), *z* = 4.92, *p* < 0.001. A significant main effect of Recognition time also emerged, with immediate recognition (*M* = 0.83, *SE* = 0.01) associated with a higher hit rate than delayed recognition (*M* = 0.73, *SE* = 0.01), *z* = 6.48, *p* < 0.001. Additionally, there was a significant main effect of Match type, such that matched trials (*M* = 0.83, *SE* = 0.01) produced a higher hit rate than unmatched trials (*M* = 0.75, *SE* = 0.01), *z* = 4.30, *p* < 0.001. All interaction terms were non-significant: the three-way interaction of Riddle type × Match type × Recognition time (*z* = −0.75, *p* = 0.451), the two-way interaction of Riddle type × Recognition time (*z* = 0.89, *p* = 0.372), the two-way interaction of Riddle type × Match type (*z* = 1.73, *p* = 0.084), and the two-way interaction of Match type × Recognition time (*z* = 0.15, *p* = 0.880).

The RT for recognition was analyzed using LMM. Results revealed a significant main effect of Recognition time, indicating that immediate recognition trials (*M* = 6.93, *SE* = 0.01) elicited faster response time than delayed recognition trials (*M* = 7.02, *SE* = 0.01), *t* = −3.03, *p* = 0.005. There was also a significant main effect of Match type, indicating that matched trials (*M* = 6.94, *SE* = 0.01) had a faster response time than unmatched trials (*M* = 7.00, *SE* = 0.01), *t* = −5.14, *p* < 0.001. However, the main effect of Riddle type was not significant, *t* = −0.85, *p* = 0.402. The interaction of Riddle type, Match type, and Recognition time (*t* = 0.77, *p* = 0.440), the interaction of Riddle type and Recognition time (*t* = −0.31, *p* = 0.755), the interaction of Riddle type and Match type (*t* = −1.23, *p* = 0.219), and Match type and Recognition time (*t* = −0.89, *p* = 0.376), were all not significant.

The RCJ was analyzed using an ordinal logistic regression model. Results showed that the main effects of Riddle type, *z* = −3.89, *p* < 0.001, Recognition time, *z* = −5.45, *p* < 0.001, and Match type, *z* = −6.54, *p* < 0.001, were all significant. The three-way interactions of Riddle type, Match type, and Recognition time, as well as the two-way interaction of Riddle type and Recognition time, were all not significant.

The two-way interaction between Riddle type and Match type was significant, *z* = 2.80, *p* = 0.005. Further simple effect analyses revealed that RCJ was significantly higher for the matched condition than for the unmatched condition for both semantic-rule riddles (*z* = 6.55, *p* < 0.001) and visual-rule riddles (*z* = 3.31, *p* < 0.001). In addition, RCJ was significantly higher for semantic-rule riddles than for visual-rule riddles in the matched condition (*z* = 4.51, *p* < 0.001), whereas there was no significant difference between the two in the unmatched condition (*z* = 1.33, *p* = 0.182).

The two-way interaction between Recognition time and Match type was also significant (*z* = 2.46, *p* = 0.014). Follow-up simple effect analyses showed that RCJ was significantly higher for the matched condition relative to the unmatched condition in both the immediate recognition condition (*z* = 6.17, *p* < 0.001) and the delayed recognition condition (*z* = 4.27, *p* < 0.001). Furthermore, RCJ was significantly higher for immediate recognition than for delayed recognition in both the matched condition (*z* = 6.00, *p* < 0.001) and the unmatched condition (*z* = 4.45, *p* < 0.001).

## 4. Discussion

A key contribution of the present study is to demonstrate the memory advantage of insight and to clarify the role of appropriateness in driving this effect for both semantic and perceptual insight. Previous studies have provided robust evidence for the mnemonic superiority of insight across a variety of tasks (Auble et al., 1979; J. Chen et al., 2023; Cui et al., 2021; Du et al., 2017; Kizilirmak et al., 2016a, 2019, 2021; Zhao et al., 2025). In particular, several studies have documented superior memory for solutions in matched versus unmatched conditions, demonstrating the memory advantage of appropriateness-based insight (Kizilirmak et al., 2016a, 2019, 2021; Zhang et al., 2025d). Consistent with this prior work, we replicate this effect: we observed higher “aha!” ratings (a core marker of insight) and better memory performance in the matched condition, where participants grasped the riddle’s rule (i.e., the logical connection between the riddle stem and solution)-than in the unmatched condition, where participants were unable to discern the riddle’s rule and thus experienced no insight.

Why does insight have an advantage in memory performance over non-insight? One account points to the integration of appropriate and novel associations (i.e., schema congruency) during the transformation process of (old to new) mental representations (S. Chen et al., 2021; Kizilirmak et al., 2016a, 2019; Zhao et al., 2025; Zhang et al., 2025d). According to Danek and Wiley (2020), solution correctness (correct vs. incorrect), involving appropriate associations, was found to predict solution recall. Consistently, Zhang et al. (2025d) reported better recognition performance in matched conditions—where participants can form appropriate associations—than in unmatched conditions—where such associations cannot be established. At the neural level, previous studies have found that activation in the medial prefrontal cortex, a region involved in forming appropriate associations (Milivojevic et al., 2015), predicted better memory performance (Kizilirmak et al., 2016a, 2019). The current study further supports the role of appropriate associations (Zhang et al., 2025d). Specifically, the sudden comprehension of matched problem–solution pairs involves integrating appropriate, task-relevant associations (e.g., the problem–solution associations), whereas no such valid associations can be formed when participants fail to comprehend unmatched problem–solution pairs.

Another account attributes the memory advantage of insight to “aha!” experience (Danek & Wiley, 2020; Kizilirmak et al., 2016a; Ludmer et al., 2011). Consistent with this view, Danek et al. (2013) and Danek and Wiley (2020) found that higher “aha!” ratings predicted better solution memory. Moreover, neuroimaging studies have found that the activity of the amygdala, a key region for emotional processing, predicts enhanced long-term memory for insight solution (Kizilirmak et al., 2016a; Ludmer et al., 2011). However, the current study does not support this account, as we found no significant relationships between “aha!” ratings and subsequent recognition memory (including hit rates, recognition rates and RT for recognition).

A second contribution of the present study is that we extend the memory superiority effect of insight to memory confidence judgments. Several studies have examined the impact of insight on retrospective confidence judgments (Ludmer et al., 2011; W. Shen et al., 2021; Zhang et al., 2025b), reporting that individuals exhibited greater confidence in their memory performance under insight (vs. non-insight) conditions. Consistent with these findings, we observed that participants were more confident in their recognition judgments in the matched (insight) condition than in the unmatched (non-insight) condition. Additionally, memory confidence judgment accuracy (MCJA) showed a marginal trend toward significance between the two conditions, *F* (1, 34) = 3.05, *p* = 0.090, η*_p_*^2^ = 0.08. This suggests that insightful comprehension not only boosts confidence judgments but may also facilitate the discrimination between correct and incorrect recognition decisions. However, given the marginal significance of this effect, future research is warranted to further validate these findings.

A third contribution is that we have revealed differences in memory performance between semantic and perceptual insight. Specifically, we found that insightful comprehension of the riddle’s semantic rule led to better memory performance (e.g., higher hit rate and recognition rate, shorter response times) than comprehension of the visuo-perceptual rule. Moreover, the significant effect of riddle type (semantic vs. visual) on memory performance was further corroborated by generalized linear mixed-effects models that account for random variation between materials (see Section 3.2.7). This suggests that semantic insight may yield better memory performance than perceptual insight. These findings not only replicate but also extend previous research on levels-of-processing theory (Baddeley & Hitch, 2017; Craik & Tulving, 1975; Roediger et al., 2002; Craik & Rose, 2012). Whereas most prior studies have shown that semantic encoding leads to better memory performance than perceptual encoding primarily in the context of routine learning and reading (e.g., processing semantic or visuo-perceptual stimuli; Friedman et al., 1996; Kuo et al., 2010; Nyhus & Curran, 2009; Paller et al., 1987), the present study extends this pattern to insightful learning and comprehension. Specifically, we showed superior memory performance when participants grasped the rule of higher-order riddles at the semantic level compared with the perceptual level.

Moreover, the memory difference between semantic and perceptual insight may be due to differences in the depth of representational transformation. Two lines of evidence support this view. First, a number of studies have indicated that representational transformation plays a critical role in the insight memory advantage via the integration of appropriate and novel associations (Becker et al., 2025; S. Chen et al., 2021; Kizilirmak et al., 2016a, 2019; Zhao et al., 2025; Zhang et al., 2025d). Second, previous theoretical and empirical studies have indicated that semantic encoding leads to better memory performance than perceptual encoding (Baddeley & Hitch, 2017; Craik & Tulving, 1975; Friedman et al., 1996; Kuo et al., 2010; Nyhus & Curran, 2009; Paller et al., 1987; Roediger et al., 2002; Craik & Rose, 2012). In the current study, the comprehension of visual-rule riddles involves representational transformation, which requires the integration of visuo-perceptual information, whereas comprehension of semantic-rule riddles involves representational transformation that requires the integration of semantic information. Thus, differences in the depth of representational transformation may underlie the divergent memory performance for solutions observed across the two riddle types. Future research is needed to test this account further.

Furthermore, people exhibited greater confidence judgment accuracy (MCJA) for semantic-rule riddles than for visual-rule riddles. A higher AUROC2 (Area under the Type 2 ROC Curve) indicates that individuals’ confidence judgments better discriminate between correct and incorrect solutions (Fleming & Lau, 2014). This suggests that, relative to perceptual processing, semantic encoding may boost MCJA—specifically, the ability to distinguish correct from incorrect solutions. More critically, a significant interaction emerged between riddle type and match type for retrospective confidence judgment (RCJ). Specifically, participants displayed significantly higher RCJ for semantic-level riddles than for perceptual-level riddles only when the solution matched the riddle problem. This indicates that the effect of insight level on memory confidence judgments depends on whether the solution matches the problem. Consistent with this finding, the effects of riddle type on RCJ were also corroborated by ordinal logistic regression analyses (see Section 3.2.7). Moreover, the difference in RCJ (matched trials minus unmatched trials) was larger for semantic-level riddles than for visual-level riddles. Collectively, this evidence demonstrates that semantic insight yields higher memory confidence judgments than perceptual insight.

However, several potential limitations should be noted. First, we did not observe a significant correlation between “aha!” experience and memory performance, which diverges from previous findings (e.g., Danek et al., 2013; Danek & Wiley, 2020). One possible reason for this discrepancy relates to the sensitivity of the “aha!” measurement. In the studies by Danek et al. (2013) and Danek and Wiley (2020), “aha!” experience was assessed using a continuous 0–100 scale, which offers high sensitivity. However, such a continuous measurement may be inconsistent with the all-or-none nature of “aha!” experience (Bowden & Jung-Beeman, 2007; Smith & Kounios, 1996). Following the recommendation of Bowden and Jung-Beeman (2007), we used a 3-point scale (1 = no aha; 2 = neutral; 3 = aha), which may have lower measurement sensitivity. Second, we retained “aha!” ratings for both matched and unmatched trials, even though “aha!” experiences were primarily intended for trials in which participants correctly comprehended matched items. We consider the inclusion of unmatched trials meaningful because it allowed participants to reliably indicate the absence of an “aha!” experience by selecting 1 (no “aha!”) for trials they did not comprehend. This is consistent with our observation that mean “aha!” ratings in the unmatched condition were close to 1 (*M* = 1.12, *SE* = 0.03). Moreover, including “aha!” ratings for unmatched trials did not compromise other measures and enabled us to capture rare instances where participants subjectively reported an “aha!” experience despite the lack of an objective match.

Third, in addition to the issues discussed above, self-generated insight is rare and often unpredictable in daily life. In the current study, we induced insight by presenting solutions, a method with greater practical relevance for learning and educational contexts. However, prior research has demonstrated that externally induced insight differs qualitatively from self-generated insight (Rothmaler et al., 2017). Future research would benefit from exploring whether memory performance varies across different degrees of self-generated insight.

Fourth, although the main effect of riddle type on memory performance (hit rate, recognition rate) suggests that insight yields better memory performance at the semantic level than at the perceptual level, this conclusion should be interpreted with caution, given the absence of a significant interaction between riddle type and match type for either hit rate or recognition rate. Post hoc power analyses conducted in the present study revealed high statistical power for the main effects of riddle type and match type on memory performance, including hit rate, recognition rate, and retrospective confidence judgments. In contrast, statistical power was low for the interaction effect between riddle type and match type on hit rate and recognition rate, whereas relatively high power was observed for this interaction effect on retrospective confidence judgments. Collectively, these findings suggest that while the current sample size was sufficient to detect the main effects of riddle type on memory performance with high power, a larger sample would be needed to reliably detect the interaction effect between riddle type and match type on memory performance. Given that the present findings provide preliminary empirical evidence for these effects, future studies are warranted to replicate and validate the current results.

Fifthly, the present study relied on a single experiment using Chinese riddles to investigate memory performance differences between the semantic-level and the visual-level induced insight. Thus, one concern is that the findings may be specific to Chinese orthography (logographic mapping, semantic radicals, and visual–spatial structure). To test the generalizability of the findings, future studies are suggested to replicate this effect by employing non-logographic writing systems or non-linguistic semantic versus perceptual problem sets. A further limitation concerns the interpretation of memory differences between the semantic-rule and visual-rule conditions, as the two conditions differed significantly in ratings of “aha!” experience, comprehensibility, ingenuity/novelty, appropriateness, and difficulty. Thus, caution is particularly warranted when comparing memory performance between the two conditions, even though the mixed-effects model analysis controlled for random variation across materials (see Section 3.2.7). Additionally, the fact that riddle ratings and memory performance data were collected from separate groups of participants limits the validity of mixed-effects model analysis that controls for these factors. Future research should examine the relationships between the characteristics of semantic versus perceptual riddles and memory performance within a single sample.

In conclusion, we demonstrate the memory superiority effect of induced insight, and in particular the role of associative appropriateness in this effect. Specifically, the appropriate (insight) condition was associated with better memory performance and higher memory confidence judgment than the inappropriate (non-insight) condition. Moreover, our findings suggest that induced insight at the semantic level yields superior memory and higher memory confidence judgments compared with insight at the perceptual level.

## Figures and Tables

**Table 1:** Descriptive statistics of the various indicators of the two types of riddles (*M* ± *SD*).

Riddle Variable	Semantic-Rule Riddle (*N* = 60)	Visual-Rule Riddle (*N* = 60)	*t*	*p*
Frequency of riddle’s solution (%)	0.05 ± 0.09	0.03 ± 0.04	−0.85	0.396
“aha!” experiences	3.57 ± 0.4	3.34 ± 0.37	−3.28	0.001
Comprehensibility	3.83 ± 0.53	4.14 ± 0.62	2.93	0.004
Ingenuity/novelty	3.77 ± 0.43	3.63 ± 0.36	−2.00	0.047
Appropriateness	3.72 ± 0.48	4.00 ± 0.64	2.67	0.009
Difficulty	0.93 ± 0.11	0.59 ± 0.32	−7.86	<0.001
Semantic conventionality	3.51 ± 0.49	3.47 ± 0.49	−0.47	0.641
Familiarity	3.58 ± 0.53	3.51 ± 0.55	−0.71	0.482
Concreteness	3.38 ± 0.46	3.35 ± 0.55	−0.29	0.775
Imagery	3.42 ± 0.36	3.38 ± 0.39	−0.57	0.572

All rating scales range from 1 (not at all) to 5 (very much).

**Table 2:** Descriptive statistics for each variable in the different conditions of the learning stage (*M* ± *SD*).

Behavioral Variable	Semantic-Rule Riddle		Visual-Rule Riddle	
	Matched	Unmatched	Matched	Unmatched
Solution rate	0.05 ± 0.09	0.06 ± 0.11	0.11 ± 0.12	0.12 ± 0.15
Comprehension rate	0.64 ± 0.21	0.12 ± 0.10	0.77 ± 0.14	0.11 ± 0.11
RT for comprehension judgment	2740.35 ± 449.79	2756.26 ± 534.32	2434.09 ± 461.48	2765.55 ± 537.38
“aha!” experiences	2.78 ± 0.22	1.13 ± 0.17	2.70 ± 0.29	1.11 ± 0.17

**Table 3:** Descriptive statistics of memory performance in different conditions (*M* ± *SD*).

Memory Variables	Recognition Time	Semantic-Rule Riddle		Visual-Rule Riddle	
		Matched	Not Matched	Matched	Not Matched
Hit rate	Immediate	0.92 ± 0.13	0.85 ± 0.15	0.83 ± 0.11	0.79 ± 0.19
	Delayed	0.87 ± 0.15	0.76 ± 0.21	0.76 ± 0.22	0.66 ± 0.20
Recognition rate	Immediate	0.74 ± 0.18	0.67 ± 0.18	0.65 ± 0.19	0.61 ± 0.23
	Delayed	0.54 ± 0.21	0.43 ± 0.2	0.43 ± 0.23	0.34 ± 0.24
RT for recognition	Immediate	1044.84 ± 252.23	1185.91 ± 311.39	1082.42 ± 240.65	1151.46 ± 276.71
	Delayed	1159.13 ± 350.37	1262.80 ± 354.93	1165.02 ± 315.37	1295.33 ± 379.34
Retrospective confidence judgment	Immediate	4.84 ± 0.25	4.51 ± 0.37	4.68 ± 0.35	4.50 ± 0.47
	Delayed	4.58 ± 0.48	4.28 ± 0.52	4.32 ± 0.53	4.22 ± 0.56
Memory confidence judgment accuracy	Immediate	0.75 ± 0.09	0.73 ± 0.09	0.71 ± 0.10	0.70 ± 0.11
	Delayed	0.70 ± 0.10	0.68 ± 0.10	0.67 ± 0.12	0.66 ± 0.12

**Table 4:** Correlations between “aha!” experiences and memory performance.

Conditions	r and *p* Value	Hit Rate	Recognition Rate	RT for Recognition	RCJ	MCJA
Immediate semantic rule & matched	r	−0.13	−0.07	0.23	−0.18	0.21
	*p*	0.475	0.70	0.18	0.31	0.23
Immediate visual rule & matched	r	0.19	0.08	−0.01	0.15	0.03
	*p*	0.284	0.67	0.95	0.38	0.88
Delayed semantic rule & matched	r	−0.21	−0.02	0.11	−0.01	0.08
	*p*	0.237	0.92	0.52	0.97	0.63
Delayed visual rule & matched	r	−0.25	−0.13	0.02	0.14	0.04
	*p*	0.143	0.46	0.92	0.41	0.81

RT: Reaction times; RCJ: Retrospective confidence judgment; MCJA: Memory confidence judgment accuracy.

## Data Availability

The data and materials are available at the website: https://doi.org/10.6084/m9.figshare.28094135.v1, and the experiment was not preregistered.
